# Unraveling the Genetic Blueprint of Doxorubicin-Induced Cardiotoxicity Through Systems Genetics Approaches

**DOI:** 10.21203/rs.3.rs-6224399/v1

**Published:** 2025-04-15

**Authors:** Buyan-Ochir Orgil, Akhilesh K. Bajpai, Neely Alberson, Morgan Lander, Batsaikhan Enkhzul, Hugo R. Martinez, Jeffrey A. Towbin, Lu Lu, Enkhsaikhan Purevjav

**Affiliations:** University of Tennessee Health Science Center; University of Tennessee Health Science Center; University of Tennessee Health Science Center; University of Tennessee Health Science Center; University of Tennessee Health Science Center; The University of Texas at Austin; University of Tennessee Health Science Center; University of Tennessee Health Science Center; University of Tennessee Health Science Center

## Abstract

**Background:**

Anthracycline-induced cardiotoxicity (ACT) is a significant concern for cancer survivors. The genetic basis of ACT remains unclear because of the impact of lifestyle and environmental factors in human studies. This study employs a murine genetic reference population (GRP) of BXD recombinant inbred strains, derived from DBA/2J (D2) and C57BL/6J (B6) crosses, to map quantitative trait loci (QTLs) linked to doxorubicin (DOX)-induced cardiotoxic phenotypes through systems genetics approaches.

**Methods:**

To model variability in ACT, 58 BXD strains and parental B6 and D2 mice (N ≥ 4 mice/sex, 3–4 months old) underwent an intraperitoneal injection of DOX (20 mg/kg). Survival and body weight (BW) were monitored for 10 days. Echocardiography was performed before treatment and on Day 5 post-treatment. Genetic mapping and Mendelian randomization (MR) analyses were used for identifying QTLs and candidate genes associated with DOX-induced traits and severity.

**Results:**

Parental B6 strain had 60% survival, whereas only 24% of D2 mice survived on Day 10. Among BXD strains, median survival varied, with BXD77 showing the lowest at four days. Echocardiography revealed restrictive dysfunction and a small-heart phenotype resembling “Grinch syndrome” observed in ACT patients. Significant QTLs on Chromosome 10 (86–94 Mb), Chromosome 19 (52.5–54.2 Mb) and on Chromosome 14 (103–120 Mb) were associated with the survival, mean BW loss, and left ventricular (LV) volumes and ejection fraction (EF%), respectively. MR analysis identified significant causal associations between the genes implicated in BW loss (*ADD3*, *HSPA12A*, *SLC18A2*, *PDZD8*, *DUSP5*, *CASP7*) as well as EF% and LV volumes (*GPC6*, *UGGT2*, *SLAIN1*, *POU4F1*, *MBNL2*) in BXD mice post-DOX and heart failure (HF) outcomes in humans.

**Conclusions:**

Survival, BW loss, and echocardiography parameters considerably varied among DOX-treated BXDs, suggesting significant influence of genetic background on expression of those traits. Several candidate genes that may modulate ACT susceptibility and HF were identified, providing a foundation for genetic-based risk stratification and therapeutics in cardio-oncology.

## Introduction

### Background:

Advances in cancer treatment have resulted in a five-year survival rate exceeding 85% for pediatric, adolescent, and young adult cancer patients. As a result, the number of long-term cancer survivors in the United States surpassed 19 million in 2024 [1, 2]. Nevertheless, anthracycline-induced cardiotoxicity (ACT) has emerged as a significant cause of late morbidity and mortality, ranking just behind cancer relapse and secondary malignancies. ACT can present as heart failure (HF), arrhythmias, sudden cardiac death, and cardiomyopathies, with its occurrence increasing among long-term survivors [3]. Anthracyclines such as doxorubicin, daunorubicin, epirubicin, and idarubicin exert irreversible myocardial damage, leading to high mortality rates among affected patients [4–6]. The risk of HF reaches 50% in patients receiving cumulative DOX doses exceeding 500 mg/m^2^ [7–9]. Furthermore, exposure to anthracyclines at a young age, combined with chest-directed radiation, exacerbates cardiovascular risk across the lifespan [3, 10–12]. A key clinical challenge is the inter-individual variability in susceptibility to ACT. While some patients tolerate high anthracycline doses without cardiotoxic effects, others develop severe cardiac dysfunction even at low doses [13]. Despite rigorous studies, the susceptibility to ACT remains incompletely understood. At the same time, recent advances in cardio-oncology have led to the hypothesis that genetic variations may contribute to an individual’s susceptibility to acute and chronic ACT [4, 14, 15]. This differential susceptibility underscores the need for genetic investigations to identify high-risk individuals. Genome-wide association studies (GWAS) have highlighted potential genetic contributors, yet their explanatory power remains limited due to low allele frequencies, environmental variability, and small cohort sizes. In addition, variable lifestyles, diets, other comorbidities, and the environment create more complexity in humans. Numerous animal studies are employed to understand the genetics underlying ACT vulnerability in cancer patients and survivors. However, traditional animal models rely on a single-genotype or engineered on a fixed genetic background, limiting their applicability to human genetic diversity. Therefore, their widespread use for population-based research persists [16].

To overcome these limitations, BXD family of recombinant inbred (RI) mice offers a powerful alternative for studying ACT susceptibility [17]. This mammalian genetic reference population (GRP) of RI mice developed by intercrossing the parental C57BL/6J (B6) and DBA/2J (D2) strains recapitulates the genetic diversity of human populations [18]. Notably, B6 parent, an “industry standard” mouse for genetic studies, has a normal heart, whereas D2 parent displays hypertrophy and fibrosis [19], making the BXD family an ideal platform for dissecting genetic predisposition to ACT [20]. Use of BXD GRPs bred and fed in laboratory conditions can point to the gene variations and genetic mechanisms because the mouse and human genomes are more than 90% syntenic.

Understanding genetic susceptibility to ACT can inform patient stratification, preventive measures, and personalized treatment strategies. This study was driven by the major hypothesis that differential susceptibility to acute and chronic ACT is associated with an individual’s genetic background, including gene variations and epistatic (gene-gene) interactions. To test, we collected survival, body weight (BW) loss, and echocardiography parameters from a large GRP of BXD mice treated with doxorubicin (DOX), identified quantitative trait loci (QTLs) and candidate genes within significant QTLs that modulate ACT traits and severity using systems genetics approaches, and validated findings for the causal relationship with the genes for HF risks and outcomes in humans.

## Methods

### Mouse BXD family

Fifty-eight BXD strains along with B6 and D2 parental lines (N≥4 mice/sex/strain, aged 3-4 months) were utilized. Mice were maintained in the 12-hour light/12-hour dark cycle on a standardized chow diet (6% kcal/fat, 20% protein, 74% carbohydrate). Experimental procedures were approved by the Institutional Animal Care and Use Committee (IACUC) at the University of Tennessee Health Science Center (UTHSC).

### Doxorubicin Treatment and Monitoring

To model ACT variability, mice received a single intraperitoneal injection of DOX (Sandoz Canada Inc) at the 20 mg/kg dosage. Survival and BW were monitored daily for 10 days. BW loss of 20% was used as the experimental endpoint. The mice were euthanized on Day 10.

### Echocardiography in mice

Serial 2-dimentional transthoracic echocardiography was performed in mice at baseline (Day -1) and Day 5 post-DOX treatment. The mouse chests were treated with a chemical hair remover to reduce ultrasound attenuation one day before echocardiography. Before the test, the mice were sedated with 1% oxygenated isoflurane anesthesia, and normothermic core temperatures were maintained using a heated platform set to 37°C. Cardiac function was then measured using B-mode, M-mode, and color Doppler with a 30-MHz transducer using Vevo2100 Micro-Imaging System (VisualSonics Inc., Toronto, Canada). Measurements of ventricular dimensions, wall thickness, and Doppler parameters, along with their analysis, were performed by two investigators who were blinded to BXD data.

### Murine heart gene expression data

Gene expression data was generated from snap-frozen heart tissues collected from BXD mice aged 29 weeks, fed a chow diet, fasted overnight and euthanized under isoflurane anesthesia as described previously [21, 22]. These data were generated through our collaborative efforts at the UTHSC and are publicly accessible via GeneNetwork (http://genenetwork.org/) with accession #GN485 [EPFL/LISP BXD CD Heart Affy Mouse Gene 2.0 ST Gene Level (Jan14) RMA]. To obtain heart gene expression, we selected Affymetrix Mouse Gene 2.0 ST microarray results from 58 male and female BXD strains and their parental B6 and D2 strains used in this study.

### Array profiling and data analysis

Briefly, raw microarray data files were first RMA (robust multichip array) normalized [23], then log2 transformed, and Z normalized as previously described [24]. Additionally, instead of maintaining a mean of zero and a standard deviation of one, we adjusted to a mean of 8 and increased the spread with a standard deviation of 2 (2Z + 8 normalization). This adjustment was made to eliminate negative values.

### Quantitative trait loci (QTL) mapping to mouse genome

All traits (BW loss, mortality, heart mass, and cardiac function) were used for genetic mapping and analyzed using the WebQTL module on our GN website as previously described [25–27]. Likelihood ratio statistics (LRS) scores were computed using Haley-Knott equations [28] to evaluate the relationships between traits and specific genotype markers across murine genome. Genome-wide significance thresholds for each QTL were defined based on 10,000 to 1,000,000 permutations of trait data [29]. All analyses were performed separately and jointly for male and female mice. The candidate genes modulating DOX-induced ACT traits in QTL and eQTL regions were narrowed down using multi-criteria approach.

### Candidate genes identification

We utilized a 2-LOD genetic interval to identify potential candidate genes. A multi-criteria system with scores ranging from 0 to 10 was employed to prioritize the genes in the selected QTL region as follows: 1) Coding variants such as non-synonymous, frame shift, and stop gain between B6 and D2, using our previously generated whole genome sequencing (WGS) data of the two parental strains [30]; 2) Genome-wide association studies for cardiovascular diseases or 3) heart failure collected from the GWAS Catalog database [31]; 4) *Cis*-regulation in BXD heart, where genes located within +/− 5 Mb of the peak QTL were considered *cis*-regulated; 5) Differentially expressed genes (DEGs) between heart failure and control participants. The GEO dataset GSE120895 [32] containing HF and standard heart samples was analyzed to identify the DEGs with adjusted p<0.05 and absolute fold change >1.5; 6) Expression in BXD heart with > 2 TPM; 7) Functional relevance in the heart based on genes collected from Mouse Genome Informatics (MGI, http://www.informatics.jax.org/) [33], International Mouse Phenotyping Consortium (IMPC, http://www.mousephenotype.org/) [34], and Rat Genome Database (RGD, www.rgd.mcw.edu) [35] as detailed in our previous article [36]; and 8) Causal significance determined by the Mendelian Randomization (MR) test. The first six criteria received a score of 1 each, whereas the last two were assigned a score of 2 each based on their overall significance. Genes with a minimum score of 30% were then selected for further analysis.

### Gene pathway analysis

To understand the biological processes and molecular mechanisms underlying ACT and to prioritize DOX-related candidate genes based on their role in critical biological processes, we performed gene pathway analysis for DEGs between untreated controls and DOX groups. The list of DEGs was uploaded to the WebGestalt website (http://www.webgestalt.org/option.php) for gene pathway analysis, which employs a hypergeometric statistical test to produce adjusted (*adj*) P values and enrichment ratios. Annotations with a minimum overlap of 5 genes and an FDR < 0.05 (Benjamini and Hochberg correction) were deemed statistically significant.

### Mendelian randomization analysis

We utilized 2-LOD interval genes for MR analysis to investigate the causal relationship between these genes and the risk of HF. The results from this analysis were then considered for the prioritization of candidate genes. Expression QTLs (eQTLs) in heart tissues [coronary artery, heart atrial appendage, and heart left ventricle (LV)] along with their effect sizes were gathered from the GTEx Portal (https://www.gtexportal.org/home/downloads/adult-gtex/qtl) [37]. GWAS summary statistics for outcomes related to HF were sourced from the IEU OpenGWAS project [38]. The following outcomes of interest were included: ebi-a-GCST009541, ebi-a-GCST90018806, finn-b-I9_HEARTFAIL, finn-b-I9_HEARTFAIL_ALLCAUSE, finn-b-I9_HEARTFAIL_AND_ANTIHYPERT, finn-b-I9_HEARTFAIL_AND_HYPERTCARDIOM, finn-b-I9_HEARTFAIL_AND_OVERWEIGHT, finn-b-I9_HEARTFAIL_EXNONE, finn-b-I9_HEARTFAIL_NS, ukb-d-HEARTFAIL, ukb-d-I50, ukb-d-I9_HEARTFAIL, ukb-d-I9_HEARTFAIL_NS, ukb-e-428_CSA, bbj-a-109, ebi-a-GCST90018586. MR analysis was conducted using the TwoSampleMR R package [38]. We employed the inverse-variance weighted method when there was more than one eQTL, while the Wald ratio was used to assess statistical significance in single eQTL.

### Statistical analysis

Data from individual mice were used to calculate the group average, and the averaged data were then used to compare among groups. The Student’s *t*-test, two-way ANOVA, or repeated-measures ANOVA over time were utilized to analyze BW and echocardiography parameters using GraphPad. At least four mice/sex from each group were studied, and a P value < 0.05 was considered significant. Pearson’s correlation analysis was used to identify relationships between baseline and post-DOX phenotypes and the expression of genes in the heart. Phenotype-correlated genes with P < 0.05 were selected for further studies and the DeSeq2 software was used to identify DEGs between baseline and post-DOX groups. Statistical significance was evaluated based on the P-value and false discovery rate (FDR). Genes with an *adjusted* P < 0.05 were considered significant DEGs and used for further systems genetics analysis.

## Results

### QTL on Chromosome 10 mean is significantly associated with survival in DOX-treated BXD strains

**Figure 1:**
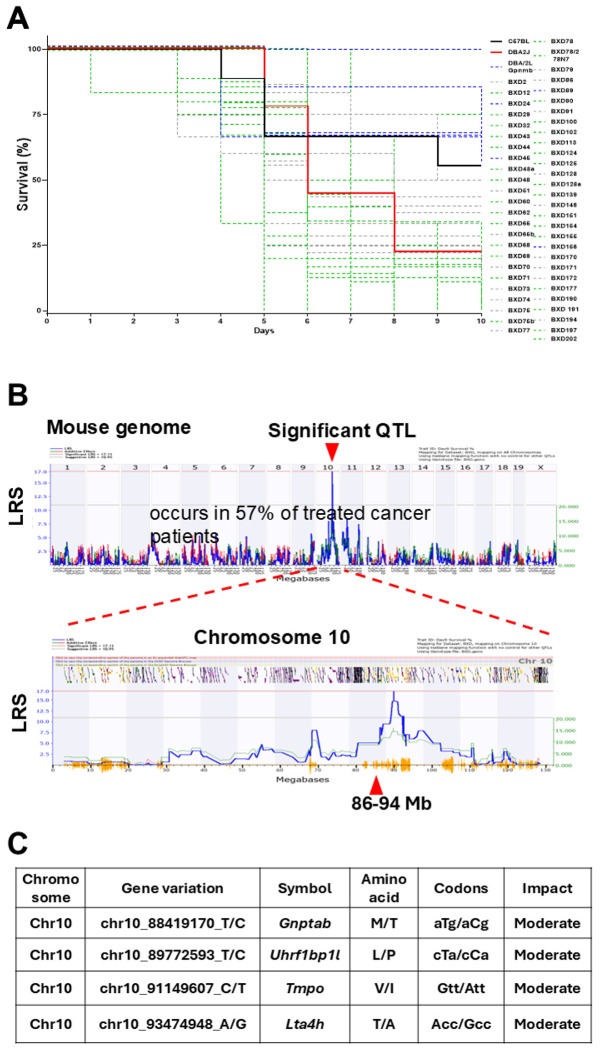
The survival rates of BXD mice undergoing DOX treatment and the genetic mapping of these survival rates to the mouse genome. **A.** The Kaplan-Meier curve illustrates the varying survival rates to DOX (Y-axis, %) among BXD strains. The X-axis represents days after DOX administration. Parental mice, B6 (black line) and D2 (red line), are indicated. The blue dashed lines denote BXD strains with longer survival rates than control B6 mice, while the green dashed lines represent BXD strains with shorter survival than D2 parental mice. **B.** Manhattan plots display a significant 86-94 megabases (Mb) QTL on Chr10 (arrowhead) associated with survival rates from Day 6 to Day 10 in BXDs treated with DOX. The X-axis represents the chromosomal position in Mb on the mouse genome, while the Y-axis shows the peak LRS (likelihood ratio statistics) score. **C.** A list of genetic variations in the top candidate genes within the Chr10 QTL identified that have genetic variations among BXD strains.

Varied survival and median survival rates were observed among DOX-treated BXDs (Figure 1A). Parental B6 mice (black line) demonstrated a mean 10-day survival rate of 60%, while only 24% of D2 mice (red line) survived for 10 days after DOX treatment. Of the 58 BXD strains tested, six strains (BXD24, 45, 74, 89, 156, 177; blue lines) exhibited higher survival rates than B6 controls, indicating a resilience (tolerance) to DOX. Other 52 BXD strains had a mean median survival of 6±1 days, showing higher mortality rates than the B6 parent. Among those strains, 26 BXDs marked by green lines displayed lower survival rates than D2 strains. Among them, BXD77, 12, 32, 65, 75b, and 78 had the lowest median survival of 4 to 5 days, followed by BXD29, 43, 62, 69, 78, 100, 102, 113, 151, and 194 (5.4 to 6 days), suggesting that these BXDs are more susceptible (vulnerable) to DOX. Notably, the highly varied survival rates among BXDs indicated significant effects of genetic background on severity and survival traits in response to DOX. Additionally, we conducted genetic mapping to identify QTLs associated with DOX-related phenotypes observed among BXDs using the WebQTL tools available on our GN website and detected a significant QTL linked to survival rates from Day 6 to 10 among DOX-treated BXDs. This QTL is at 86–94 Mb on Chr 10 (Figure 1B). Moreover, we refined the candidate genes within this QTL for further validation using a multi-criteria approach as described previously [21, 39], which identified *Gnptab*, *Slc25a3*, *Uhrf1bp1l*, and *Chpt1* within the Chr10 QTL as top candidate genes with a minimum 30% score. Further, genotype analysis clarified that *Gnptab* and *Uhrf1bp1l* genes had missense variants with moderate effects segregated among BXD strains (Figure 1C).

### Chromosome 19 is associated with body weight loss among DOX-treated BXD mice.

**Figure 2:**
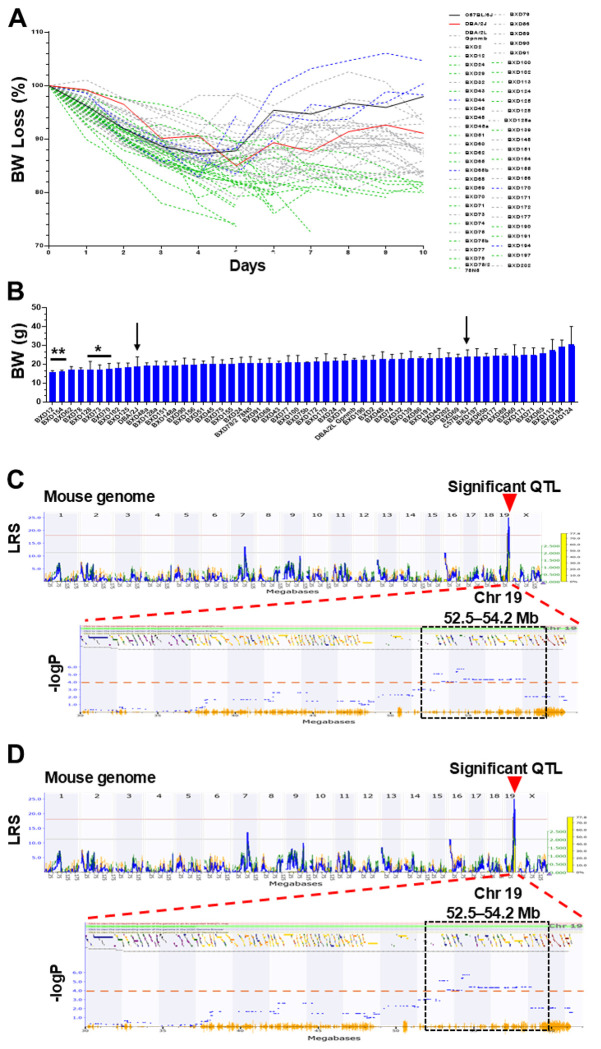
Rates of body weight (BW) loss in BXDs to DOX treatment and genetic mapping of BW loss to mouse genome. **A.** Varied rates of mean BW loss in percent (%, Y-axis) among BXDs during 10 days of DOX treatment (X-axis). The BXD strains with significantly higher BW loss (green dashed lines) compared to parental D2 (red line) mice and BXDs with lower BW loss (blue dashed lines) than B6 control mice (black line) are indicated. **B.** Values of BW in grams (g, Y-axis) on Day 10 post-DOX treatment among BXD mice (X-axis). Arrows indicate parental B6 and D2 mice. Asterisks (*=P<0.05, **=P<0.01) indicate significant differences in BW compared with control B6 strains. **C-D.** Manhattan plots showing a significant QTL of 52.4-54.2 Mb (arrowhead) on Chr19 associated with the BW loss mean in grams (g, **C**) and in percent (%, **D**) in BXDs on Day 10. Y-axis, LRS and -logP scores.

Interestingly, BW loss between parental B6 (Figure 2A, black line) and D2 (red line) mice significantly differed, reaching the maximum mean BW loss in D2 mice on D6 (14%) and BW regain was slow with only 5% increase on Day 10. In contrast, B6 mice exhibited 12% mean BW loss on Days 3-5 with sharp regain on Day 6 reaching 98% of baseline WB values on Day 10 post-DOX. The BW loss varied among BXDs following the DOX injection as well. BXD strains with significantly higher BW loss on Day 10 compared to B6 parental mice included BXD12, 154, 128, 73, and 70 (Figure 2B). In contrast, strains BXD194 and 124 showed higher BW on Days 9-10 compared to that at the baseline, suggesting that those strains are more tolerant to DOX. Of all the 10 days tested, Day 10 revealed a significant QTL on Chr19 (52.5~54.2 Mb) associated with mean BW loss (grams and %) in BXDs based on both GEMMA mapping and Haley-Knott regression methods. Further multi-score filtering identified 22 genes with a 30% score (Table 1), among which *Hspa12a*, *Rbm20*, *Adrb1*, and *Pdzd8* with a ≥5 significance score are considered the best candidate genes associated with mean BW loss on Day 10 post-DOX treatment (Figure 2C–D).

### DOX injection caused reduced left ventricular mass and restrictive dysfunction in BXDs

**Figure 3:**
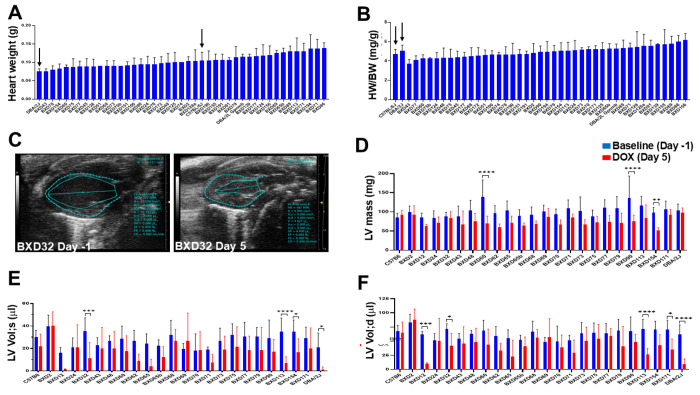
Results of cardiac morphology and echocardiographic assessment in BXDs treated with DOX. **A-B.**Results of gross heart weight (HW) analysis in BXD mice on Day 10 post-DOX. Y-axis: Values of HW in grams (g, **A**) and heart weight to body weight (HW/BW, mg/g) ratio in arbitrary units (**B**) in BXD mice (X-axis). Arrows indicate parental B6 and D2 strains. **C.**Representative echocardiographic long axis view images of BXD mouse heart at baseline (Day -1) and on Day 5 post-DOX treatment. Left ventricular (LV) walls and chamber length are indicated. **D-F.** Results of comparative echocardiography assessment at the pre- and post-DOX treatment. Y-axis: Values of LV mass (mg, **D**), LV volumes (ml) at systole (s, **E**) and diastole (d, **F**) among BXDs (X-axis) at the baseline (Day -1, blue columns) compared to Day 5 post-DOX treatment (red columns). Asterisks denote significant differences (*=P<0.05, **=P<0.01, ***=P<0.001, ****=P<0.0001) between Day -1 and Day 5.

Early detection of abnormal cardiac morphology and function in cancer patients and survivors is vital to prevent residual risks of anthracycline chemotherapy for HF and cardiac death later in life [40]. Most pediatric cancer patients treated with anthracyclines who exhibit ACT have smaller hearts characterized by a shrinking myocardial and cavity size, referred as “Grinch syndrome” [41]. This condition poses a long-term risk for young adults and older cancer survivors [8]. Heart weight (HW) and HW/BW ratio measured on Day 10 revealed no significant variations of those traits among BXDs (Figure 3A–B), suggesting concordant changes in BW and HW in mice post-DOX. Serial echocardiography performed on Day 5 post-DOX treatment in 24 BXD lines (≥4 mice/sex/strain) defined abnormal small hearts with reduced left ventricular (LV) mass (Figure 3C–D) and LV volumes at systole and diastole (Figure 3E–F) compared to the Day -1 baseline. Notably, LV mass was significantly reduced in BXD60, 90, and 154 strains, while BXD32, 113, 154, and D2 mice showed significant reductions in LV volumes, resembling the “Grinch syndrome” observed in humans and suggesting that these strains are vulnerable to DOX (**Supplementary Video**).

**Figure 4:**
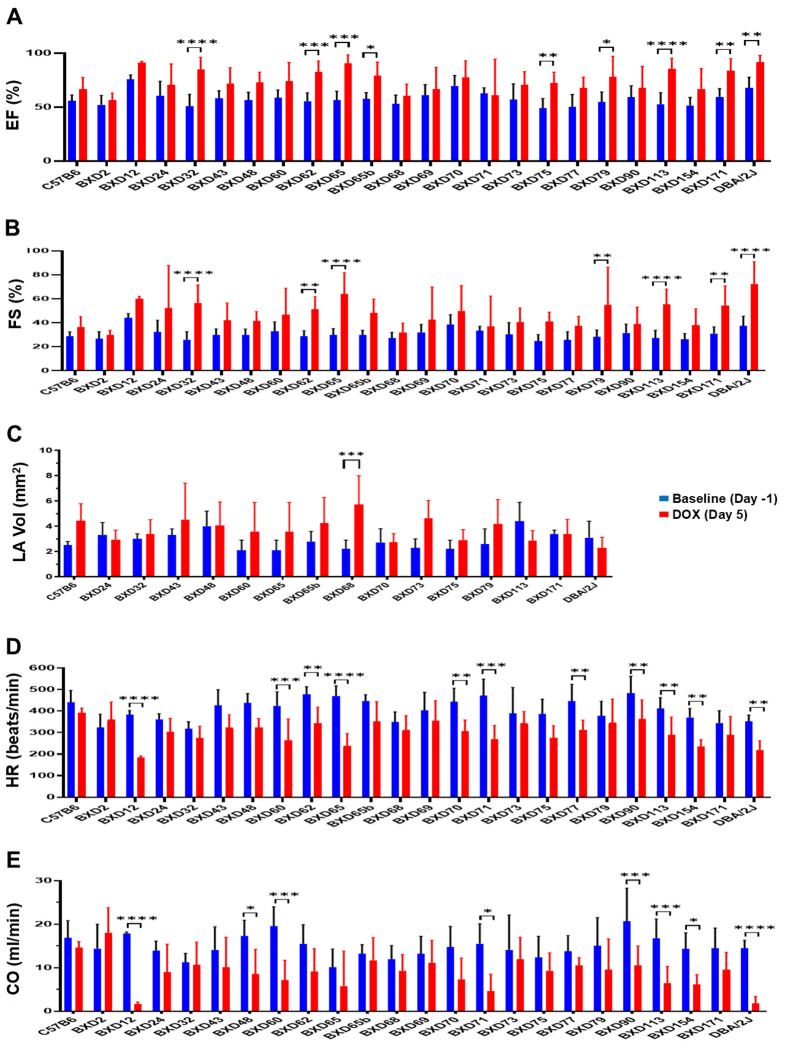
Results of echocardiographic assessment of heart function in BXDs treated with DOX. Y-axis: **A**, ejection fraction (EF%); **B,** fractional shortening (FS%); **C,** left atrial (LA) volumes (mm^2^); **D,** heart rate (HR, beats/min); **E,** cardiac output (CO, ml/min) among BXDs (X-axis) at baseline (Day -1, blue columns) compared to Day 5 post-DOX treatment (red columns). Asterisks denote significant differences between Day -1 and Day 5.

All other echocardiographic parameters, including fractional shortening (FS%), ejection fraction (EF%), heart rate (HR), and cardiac output (CO), were abnormally altered in response to DOX-treatment and varied significantly among BXDs (Figure 4 and **Supplementary Figure 1**). Although EF% and FS% markedly increased in many BXDs (Figure 4A–B) presumably due to LV restrictive physiology resulting in the increase of left atrial (LA) volume (Figure 4C), HRs were reduced (Figure 4D), resulting in CO decline in most BXDs post-DOX treatment, demonstrating reduction in an amount of blood pumped per minute (ml/min) by the heart to vital organs (Figure 4E). Some BXDs (BXD12, 48, 60, 71, 90, 154) and D2 mice presented a significant decrease in CO, suggesting that these strains have greatly diminished oxygen delivery to the tissues and are more vulnerable for developing ACT and CHF. Collectively, the significantly varied traits of cardiac morphology and function among BXDs indicated the underlying impact of genetic background on expression of all those cardiac traits to DOX treatment.

### QTL mapping of echocardiography parameters

**Figure 5:**
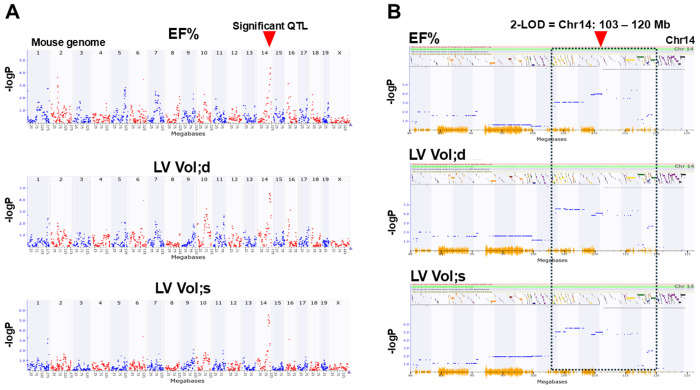
Results of GEMMA genetic mapping of echocardiography traits in BXDs in response to DOX. Manhattan plots showing a significant 103-120 Mb QTL (arrowhead): **(A)** on mouse genome (X-axis); and **(B)** on Chr14 associated with ejection fraction (EF%) and LV volumes at systole (LV Vol;s) and diastole (LV Vol;d). Y-axis indicates an -logP score.

We conducted QTL mapping for all echocardiography parameters collected sequentially on Day -1 and Day 5 post-DOX treatment. Among the echocardiography traits, EF%, LVVol;d, and LVVol;s revealed significant QTLs on Chr14 between 103-120 Mb using GEMMA genetic mapping (Figure 5). Additionally, we identified 9 genes as top candidate genes in the Chr14 QTL through our multi-score selection system (Table 2). Among those, we considered *Mycbp2*, *Abcc4* (score of 4) and *Ednrb*, *Gpr180*, *Mbnl2* with a fold change (FC>3) as strong candidate genes linked to changes in EF% and LV volumes in response to DOX due to their high levels of gene expression in BXD hearts.

Taken together, our studies in the BXD family of mice treated with DOX demonstrated varied expression of all ACT-related phenotypes (traits) tested, including survival, BW loss, cardiac function and morphology, indicating significant impacts of genetic background on these phenotypic responses to DOX treatment. We identified significant QTLs and candidate genes within the mouse genome associated ACT-related traits expressed in response to DOX, suggesting that those genes identified in murine GRP may play a role in differential susceptibility to human ACT-related phenotypes and require further causal and functional validation using MR analysis.

### Mendelian Randomization analysis

We used MR to analyze the causal associations between the 2-LOD interval genes and HF for both BW loss after 10 days of DOX treatment and echocardiographic traits (EF% and LV volumes). The 2-LOD interval related to BW loss post-DOX treatment contained 159 genes. We identified 27 eQTLs corresponding to 18 genes in the heart tissues from the GTEx database. The association of these genes with sixteen HF outcomes obtained from the IEU OpenGWAS project was assessed, yielding 202 associations, and twenty of these associations, relating to 6 genes (*ADD3*, *HSPA12A*, *SLC18A2*, *PDZD8*, *DUSP5*, *CASP7*), were statistically significant with P<0.05 (Table 3). Furthermore, the 2-LOD region associated with cardiac EF% and LV volumes traits included 211 genes. For 16 of these genes, 27 eQTLs in heart tissues were retrieved from the GTEx database. The association of these genes with HF outcomes resulted in a total of 234 associations, of which 14, corresponding to 5 genes (*GPC6*, *UGGT2*, *SLAIN1*, *POU4F1*, *MBNL2*), were statistically significant (Table 4).

## Discussion

Anthracycline-induced cardiotoxicity continues to be a significant cause of late-onset cardiovascular diseases in long-term cancer survivors, especially among youth, the elderly, and individuals with cardiovascular comorbidities [42]. However, the clinical management of patients at risk for developing ACT remains fragmented and not patient centered [43]. Clinically, identifying vulnerable patients at increased risk for ACT could enable precision medicine approaches, including dose modification of anti-cancer drugs, early cardioprotective interventions, and long-term cardiac monitoring. To elucidate the genetic determinants underlying ACT risks and severity, our study utilized a systems genetics approach in the best-characterized murine GRP of BXD RI mice derived from the parental D2 strain that exhibited natural characteristics of cardiomyopathy [19, 20]. The study results confirmed that genetic background significantly influenced variability of ACT-related traits such as the survival, BW loss, and cardiac morphology and dysfunction phenotypes among BXD mice following DOX exposure. Notably, we observed a distinctive “Grinch syndrome” phenotype in many DOX-treated BXD strains, characterized by reduced LV mass, LV volumes, and restrictive or diastolic dysfunction, mirroring clinical reports of small LV dimensions and impaired diastolic function in ACT patients [8, 41].

We also identified significant QTLs pinpointing important candidate genes within murine genomic regions modulating those ACT traits and their severity. For example, murine genes encoding proteins such as ATP-binding cassette transporter *(Abcc4)* and heat shock protein *(Hspa12a)* in the Chr19 QTL and solute carrier proteins *(Slc25a3* and *Slc18A2* on murine Chr10 and Chr19, respectively*)* have been known to be involved in cellular metabolism, mitochondrial function, epigenetic regulation, and cardiac fibrosis, aligning with findings from human studies, where specific genetic polymorphisms have been implicated in the pathogenesis of ACT [44–46]. Moreover, the study identified human ACT-susceptibility genes such as *ADD3, HSPA12A, SLC18A2, PDZD8, DUSP5, CASP7, GPC6, UGGT2, SLAIN1, POU4F1, MBNL2* that were significantly associated with HF outcomes in humans using MR analysis. Related to cancer and ACT therapeutic targets, knockdown of *PDZD8*, encoding the mitochondria-associated endoplasmic reticulum membrane protein, has shown to significantly promote anti-tumor activity of sunitinib, an inhibitor of tyrosine kinase receptor used in combination with antioxidant pterostilbene [47], while expression of *POU4F1* (Pit-Oct-Unc domain transcription factor) significantly correlated with cancer patient survival and *POU4F1* knockdown inhibited proliferation of esophageal squamous carcinoma cells [48]. RNA-binding protein MBNL2 (muscleblind-like splicing regulator 2) has shown to contribute to the post-transcriptional gene dysregulation in renal cell carcinoma [49], while loss-of-function mutations of *CASP7* (caspase 7) contributed to cancer pathogenesis, representing an important prognostic and therapeutic target for several solid cancers [50].

One of this study’s key findings of this study is an identification of *DUSP5* (dual specificity phosphatase 5) that associated significantly with BW loss in DOX-treated BXDs as well as with HF outcomes in humans, offering an intriguing potential implication for *DUSP5* in mitigating various anti-cancer drug-induced cardiotoxicities. The DUSP5 enzyme is known to dual inactivate mitogen-activated protein kinases (MAPKs) such as extracellular signal-regulated kinases (ERK1/2) that are involved in activation of downstream targets relevant in many diseases and cancers [51]. Recent development and use of inhibitors of ERK-signaling alone or in combination with revolutionary immune checkpoint inhibitors (ICI) targeting the receptors of cytotoxic T-lymphocyte-associated 4 (CTLA4) and programmed death-1 (PD1) proteins significantly improved survival of cancer patients [52]. However, these therapies are associated with multi-organ adverse events, including cardiotoxicity, HF, and ICI-induced myocarditis with high mortality rates of 16–50% in affected patients [53]. Supporting our results, further recent study has shown that *DUSP5* suppression supplemented with thyroid T3 hormone treatment reversed LV dilatation and dysfunction in mice with cardiac chronic DOX injury by increasing numbers of functional cardiomyocytes [54]. Based on these reports, our murine findings warranted further validation of identified genes in cancer patients not only undergoing anthracyclines regiment but also other anti-cancer therapies.

In summary, this study advances our understanding of the genetic basis of ACT and broadens the potential for integrating translational medicine into clinical practice. We suggest that future research leveraging large-scale human biobanks and precision medicine initiatives will be critical for translating our murine findings into clinical applications. Additionally, personalized risk prediction models using systems biology approaches by integrating genetic, clinical, multi-omics (transcriptomics, proteomics, and metabolomics) data, lifestyle and environmental factors should elucidate the molecular mechanisms linking genetic variation(s) to ACT susceptibility, enabling the early identification of high-risk individuals and guiding personalized cardioprotective strategies in cancer patients and survivors. By bridging the gap between experimental and translational genetics and medicine, our findings pave the way for improved cardioprotective and therapeutic strategies in cancer patients and survivors, ultimately reducing the burden of cardiovascular complications in this vulnerable population.

## Study limitations

Despite the strengths of our systems genetics study, several limitations must be acknowledged. First, while murine GRPs allow for controlled environmental conditions and facilitate genetic mapping, direct extrapolation to human populations with diverse environmental influences requires cautious interpretation. Therefore, genetic diversity in humans is more significant than in RI murine models bred and fed in controlled conditions, necessitating the validation of identified QTLs and candidate genes in human cohorts through GWAS or functional assays. Second, our study focused on high-dose DOX exposure, which may not fully capture the complexity of chronic low-dose anthracycline exposure in clinical settings. Our future studies will investigate dose-dependent and long-term responses to anthracyclines across different genetic backgrounds. Lastly, although a Mendelian randomization analysis has been achieved for identifying causal associations between candidate genes and the risk of HF through OpenGWAS project, a functional validation of those genes is needed to confirm causal relationships between ACT phenotypes (traits) and genetic variants and candidate gene expression through molecular biology technologies including CRISPR-Cas9 gene-editing and immunological methods.

## Figures and Tables

**Table 1.** List of genes with score of 30% associated with BM loss in BXDs to Day 10 of DOX treatment.

Symbol	Gene Description	Coding variants between B6 and D2	GWAS (heart failure)	GWAS (cardiovascular disease)	Cis-regulation in heart	DEG in Cardiomyopathy vs Control	Functional relevance in heart	Expression in BXD heart	Mendelian Randomization	Total score
***Hspa12a***	heat shock protein 12A	Nonsynonymous SNP	Y	Y	Y	--	--	3.35	Y	**7**
***Rbm20***	RNA binding motif protein 20	Nonsynonymous SNP	--	Y	--	Y	Y	6.92	--	**6**
***Adrb1***	adrenergic receptor, beta 1	--	--	Y	--	Y	Y	4.32	--	**5**
***Pdzd8***	PDZ domain containing 8	--	--	Y	--	Y	--	3.62	Y	**5**
*Pdcd4*	programmed cell death 4	--	--	--	--	Y	Y	4.49	--	4
*Shoc2*	Shoc2, leucine rich repeat scaffold protein	--	--	--	--	Y	Y	3.57	--	4
*Afap1l2*	actin filament associated protein 1-like 2	missense_variant	--	Y	--	--	Y	1.86	--	4
*Ablim1*	actin-binding LIM protein 1 solute carrier family 18 (vesicular monoamine),	Nonsynonymous SNP	--	--	Y	Y	--	6.88	--	4
*Slc18a2*	member 2	Nonsynonymous SNP	--	--	Y	--	--	1.26	Y	4
*Add3*	adducin 3 (gamma)	--	--	--	--	--	--	4.94	Y	3
*Dusp5*	dual specificity phosphatase 5	--	--	--	--	--	--	2.89	Y	3
*Bbip1*	BBSome interacting protein 1	--	--	--	--	--	y	3.59	--	3
*Adra2a*	adrenergic receptor, alpha 2a acyl-CoA synthetase long-chain family member	--	--	Y	--	--	Y	0.06	--	3
*Acsl5*	5 vesicle transport through interaction with t-	--	--	--	Y	--	Y	1.62	--	3
*Vti1a*	SNAREs 1A transcription factor 7 like 2, T cell specific, HMG	--	--	Y	--	Y	--	2.76	--	3
*Tcf7l2*	box	--	Y	Y	--	--	--	3.23	--	3
*Nrap*	nebulin-related anchoring protein	Nonsynonymous SNP	--	Y	--	--	--	8.40	--	3
*Casp7*	caspase 7	--	--	--	--	--	--	3.68	Y	3
*Nhlrc2*	NHL repeat containing 2 glial cell line derived neurotrophic factor family	--	--	Y	Y	--	--	3.83	--	3
*Gfra1*	receptor alpha 1	Splice site mutation	--	Y	--	--	--	2.63	--	3
*Pnliprp2*	pancreatic lipase-related protein 2	Nonsynonymous SNP	--	Y	Y	--	--	0.01	--	3
*Rab11fip2*	RAB11 family interacting protein 2 (class I)	--	--	--	Y	Y	--	2.30	--	3

**Comment:** The top candidate genes with a score of ≥ 5 out of 10 are highlighted in bold.

**Table 2.** List of genes with a score of 30% within the Chr14 QTL associated with cardiac function traits in BXDs to DOX treatment.

Symbol	Gene Description	Coding variants between B6 and D2	GWAS (heart failure)	GWAS (cardiovascular disease)	Cis-regulation in heart	DEG in Cardiomyopathy vs Control	Functional relevance in heart	Expression in BXD heart	Mendelian Randomization	Total score
***Mycbp2***	**MYC binding protein 2, E3 ubiquitin protein ligase**	--	--	Y	--	--	Y	2.79	--	**4**
***Abcc4***	**ATP-binding cassette, sub-family C (CFTR/MRP), member 4**	Nonsynonymous SNP	--	Y	--	Y	--	3.39	--	**4**
*Slain1*	SLAIN motif family, member 1	Nonsynonymous SNP	--	--	--	--	--	0.14	Y	**3**
*Ednrb*	endothelin receptor type B	Nonsynonymous SNP	--	--	--	Y	--	3.75	--	**3**
*Gpc6*	glypican 6	--	--	--	--	--	--	3.10	Y	**3**
*Tgds*	TDP-glucose 4,6-dehydratase	--	--	--	--	--	Y	2.29	--	**3**
*Gpr180*	G protein-coupled receptor 180	--	--	--	Y	Y	--	4.95	--	**3**
*Uggt2*	UDP-glucose glycoprotein glucosyltransferase 2	Nonsynonymous SNP	--	--	--	--	--	1.40	Y	**3**
*Mbnl2*	muscleblind like splicing factor 2	--	--	--	--	--	--	5.18	Y	**3**

**Comment:** The top candidate genes with a score of 4 out of 10 are highlighted in bold.

**Table 3:** Significant causal associations between the QTL genes linked to BW loss following Day 10 post-DOX treatment and heart failure outcomes

Gene	Outcome	No. of SNPs	Method	P value
*ADD3*	Heart failure || id:ebi-a-GCST009541	1	Wald	0.01488
*HSPA12A*	Heart failure || id:ebi-a-GCST009541	2	IVW	0.04871
*SLC18A2*	Congestive heart failure || id:bbj-a-109	2	IVW	0.03255
*SLC18A2*	Chronic heart failure || id:ebi-a-GCST90018586	2	IVW	0.00311
*SLC18A2*	Chronic heart failure || id:ebi-a-GCST90018806	2	IVW	0.001679
*PDZD8*	Congestive heart failure || id:bbj-a-109	2	IVW	0.04751
*PDZD8*	Chronic heart failure || id:ebi-a-GCST90018586	2	IVW	0.004832
*PDZD8*	Chronic heart failure || id:ebi-a-GCST90018806	2	IVW	0.002459
*DUSP5*	All-cause Heart Failure || id:finn-b-I9_HEARTFAIL_ALLCAUSE	1	Wald	0.007077
*DUSP5*	Heart failure and antihypertensive medication || id:finn-b-I9_HEARTFAIL_AND_ANTIHYPERT	1	Wald	0.005666
*DUSP5*	Heart failure and bmi 25plus || id:finn-b-I9_HEARTFAIL_AND_OVERWEIGHT	1	Wald	0.007237
*DUSP5*	Heart failure, not strict || id:finn-b-I9_HEARTFAIL_NS	1	Wald	0.007237
*DUSP5*	Heart failure || id:ukb-d-HEARTFAIL	1	Wald	0.003835
*DUSP5*	Diagnoses - main ICD10: I50 Heart failure || id:ukb-d-I50	1	Wald	0.002606
*DUSP5*	Heart failure,strict || id:ukb-d-I9_HEARTFAIL	1	Wald	0.003835
*DUSP5*	Heart failure, not strict || id:ukb-d-I9_HEARTFAIL_NS	1	Wald	0.00383
*CASP7*	Congestive heart failure || id:bbj-a-109	3	IVW	0.000525
*CASP7*	Heart failure || id:ebi-a-GCST009541	2	IVW	0.00137
*CASP7*	Chronic heart failure || id:ebi-a-GCST90018586	3	IVW	7.97E-05
*CASP7*	Chronic heart failure || id:ebi-a-GCST90018806	3	IVW	0.00223

Comment: IVW, Inverse variance weighted

**Table 4:** Significant causal associations between the QTL genes linked to echocardiography EF% and LV volume traits and heart failure outcomes

Gene	Outcome	No. of SNPs	Method	P value
*MBNL2*	All-cause Heart Failure || id:finn-b-I9_HEARTFAIL_ALLCAUSE	1	Wald	0.02909
*MBNL2*	Heart failure and bmi 25plus || id:finn-b-I9_HEARTFAIL_AND_OVERWEIGHT	1	Wald	0.02553
*MBNL2*	Heart failure, not strict || id:finn-b-I9_HEARTFAIL_NS	1	Wald	0.02553
*POU4F1*	Heart failure || id:ebi-a-GCST009541	1	Wald	0.009118
*POU4F1*	Heart failure || id:ukb-d-HEARTFAIL	1	Wald	0.04485
*POU4F1*	Diagnoses - main ICD10: I50 Heart failure || id:ukb-d-I50	1	Wald	0.04475
*POU4F1*	Heart failure,strict || id:ukb-d-I9_HEARTFAIL	1	Wald	0.04485
*POU4F1*	Heart failure, not strict || id:ukb-d-I9_HEARTFAIL_NS	1	Wald	0.04485
*SLAIN1*	Chronic heart failure || id:ebi-a-GCST90018806	1	Wald	0.04101
*UGGT2*	Congestive heart failure || id:bbj-a-109	3	IVW	0.04843
*UGGT2*	Heart failure || id:ukb-d-HEARTFAIL	3	IVW	0.02503
*UGGT2*	Heart failure,strict || id:ukb-d-I9_HEARTFAIL	3	IVW	0.02503
*UGGT2*	Heart failure, not strict || id:ukb-d-I9_HEARTFAIL_NS	3	IVW	0.02503
*GPC6*	Heart failure || id:ebi-a-GCST009541	2	IVW	4.48E-05

Comment: IVW, Inverse variance weighted

## Data Availability

The expression data for heart tissues from BXD mice are available on the GeneNetwork (http://genenetwork.org/) with accession #GN485 [EPFL/LISP BXD CD Heart Affy Mouse Gene 2.0 ST Gene Level (Jan14) RMA]. All other data presented are available either in the main manuscript or in the Supplementary Material.
